# Effects of an 8-week high intensity interval training (HIIT) and ashwagandha supplementation on aerobic capacity, muscle oxygenation and haematological parameters in healthy men

**DOI:** 10.5114/biolsport.2025.147453

**Published:** 2025-02-04

**Authors:** Jówko Ewa, Andrzej Klusiewicz, Kinga Rębiś, Barbara Długołęcka, Małgorzata Charmas, Igor Cieśliński

**Affiliations:** 1 Józef Piłsudski University of Physical Education in Warsaw, Faculty of Physical Education and Health, Biała Podlaska, Department of Physiology and Biochemistry, Poland; 2 Institute of Sport – National Research Institute in Warsaw, Department of Physiology, Poland; 3 Józef Piłsudski University of Physical Education in Warsaw, Faculty of Physical Education and Health, Biała Podlaska, Department of Sports and Training Sciences, Poland

**Keywords:** Herbal supplement, High intensity interval training, Rowing ergometer, Maximal graded exercise test, Maximal oxygen uptake, Anaerobic threshold, Near-infrared spectroscopy, Hematological parameters

## Abstract

Ashwagandha extract is an herbal dietary supplement with suggested health properties and the potential to improve physical performance. However, there are still few well-controlled studies confirming improvements in aerobic capacity as a result of supplementation, as well as the mechanisms responsible for this effect. The purpose of this randomized, double-blind, placebo-controlled study was to evaluate the effects of ashwagandha supplementation (600 mg/day) on aerobic capacity, muscle oxygenation and resting blood haematological parameters in healthy male non-athletes ’participants undergoing 8 weeks of HIIT. The training was performed on a rowing ergometer (3 sessions per week, 5–7 sets of 1.5 minutes in each session, at a load of 85–95% of maximum aerobic power, and with rest intervals between sets of 1.5 minutes at a power output of 70 W). Aerobic capacity was determined during a maximal graded exercise test on rowing ergometer, performed before and after the 8-week intervention. During this test, muscle oxygenation was monitored using a near-infrared spectroscopy monitor. The two-factor ANOVA (2 × 2; time × group) revealed no main effect of group and interaction of time and group in the aerobic capacity or haematological parameters (P > 0.05). In turn, as a result of training, both placebo (n = 16) and ashwagandha (n = 17) groups showed large significant improvements in aerobic capacity parameters, i.e. test time, maximal aerobic power and anaerobic threshold (main effect of time, P = 0.00001, post-hoc differences within group; pre-post, P < 0.001; with η^2^ amounted 0.60–0.78), with less pronounced changes in maximal oxygen uptake (in absolute terms, main effect of time, P = 0.019; η^2^ = 0.16). Furthermore, significant main effect of time and interaction of time and group in some parameters of muscle oxygen utilisation (P < 0.05; η^2^: 0.15–0.19) were found, and post-hoc analyses showed significant intragroup (pre-post) differences (improvements) in the placebo group (P < 0.05), but not in the ashwagandha group (P > 0.05). In conclusion, in healthy men participating in the 8-week HIIT, ashwagandha supplementation (at dose 600 mg daily) does not appear to affect hematological status or offer additional benefits in aerobic capacity over those observed under training. Moreover, the inconclusive effect of ashwagandha on training-induced changes in muscle oxygenation needs to be verified in further studies.

## INTRODUCTION

Ashwagandha (*Withania somnifera*) is a plant whose roots are used in traditional Eastern medicine to increase resistance to physical and mental stress. The active constituents of ashwagandha extracts that may be responsible for the health properties are believed to include steroidal lactones (withanolides) and flavonoids, as well as saponins and sterane derivatives. A number of studies have reported the potential of these substances to regulate adrenal functions, among others, as well as effects on haematological indices [1]. An increase in blood haemoglobin (Hb) concentration and red blood cell count (RBC) have been pointed to as a key mechanism to explain the increase in maximal oxygen uptake (${\dot{\text{V}}\text{O}}_{2\max}$) after ashwagandha supplementation. As suggested, the increase in RBC and Hb may lead to an increase in the blood’s ability to transport oxygen to exercising muscles, thereby increasing aerobic capacity [2].

There are several research papers in the available scientific literature on the effects of ashwagandha supplementation on exercise performance, conducted mainly in Indian population. The results of meta-analyses based on those studies indicated an improvement in aerobic capacity as a result of ashwagandha administration, both in untrained and trained individuals [1, 3]. However, it should be noted that in most of the papers included in the meta-analysis, the assessed index of aerobic capacity, ${\dot{\text{V}}\text{O}}_{2\max}$, was determined by indirect methods, or the data presented are difficult to interpret due to the lack of precise information on the method of measuring ${\dot{\text{V}}\text{O}}_{2\max}$ and monitoring training loads. Therefore, studies aimed at evaluating the chronic effects of ashwagandha administration on the aerobic capacity of non-athletes undergoing fully supervised physical training are warranted. High-intensity interval training is worthy of attention because of the physical and physiological benefits this method of training provides to untrained individuals, as well as to recreational exercisers or competitive athletes [4, 5].

HIIT usually refers to “repeated series of exercises that occur at power output or speed in the high-intensity zone that takes place between the second ventilatory threshold and ${\dot{\text{V}}\text{O}}_{2\max}$[6]. HIIT training aims to load the body’s functional systems to a greater degree than is required during traditional endurance training and is considered more effective in increasing ${\dot{\text{V}}\text{O}}_{2\max}$ compared to continuous endurance training [4]. Furthermore, there are also reports indicating that HIIT is superior to traditional endurance training with regard to improving a number of health markers in both healthy and chronically ill populations [7]. In addition, due to the shortened duration of the training unit, HIIT is considered an alternative, effective form of training to improve physical capacity while reducing the overall time spent on training, which is especially important given that lack of time is a commonly cited barrier to exercise participation [7]. The results of previous studies indicate that even a few HIIT sessions may be sufficient to improve exercise capacity [8]. In that study, in previously untrained young adults, six HIIT sessions (cycling) over two weeks resulted in an increase in ${\dot{\text{V}}\text{O}}_{2\max}$, peak power output and time-trial performance, and these improvements occurred concomitantly with an increase in skeletal muscle respiratory capacity [8].

Notably, the use of a rowing ergometer in HIIT training allows for the involvement of almost all muscle parts (both lower and upper body) and also the incorporation of resistance exercise elements [9]. Simulated rowing is therefore recommended as an effective way to achieve optimal health benefits and prevent chronic diseases [10]. At the same time, training on a rowing ergometer is safe for subjects (low injury rate) and is widely practiced among both young and elderly people [9].

In the study, a mobile measuring device that uses near-infrared spectroscopy (NIRS) to assess the degree of myoglobin oxygenation in the muscle cytoplasm and haemoglobin in the blood vessels of the muscle microcirculation (muscle oxygen saturation, SmO_2_) was used to assess the body’s exercise response [11]. In contrast to ${\dot{\text{V}}\text{O}}_{2\max}$ which assesses global cardio-respiratory capacity, SmO_2_ is a local measurement of the cell’s aerobic metabolism, which can be potentially influenced by ashwagandha supplementation. Many authors claim that muscle oxygenation is a simple, safe, and fast way to both assess exercise capacity in short-term maximal contractions [12, 13] and to determine so-called training intensity zones [14–16].

In addition, modern oximeters make it possible to perform tests during competitive efforts under natural conditions in various sports [17, 18], and SmO_2_ measurements can also be helpful for determining training load (including exercise intensity, resting rate as well as training duration) [19–21]. Devices using NIRS technology most often monitor a variety of indicators, but in studies of athletes, muscle tissue oxygenation (SmO_2_) and total haemoglobin (tHb) are mainly analyzed. The SmO_2_ value reflects changes in muscle oxygen delivery and consumption, expressed as the ratio of oxygenated haemoglobin (oxyhemoglobin, HbO_2_) and myoglobin (oxymyoglobin, MbO_2_) to total haemoglobin. The tHb index is used to assess local blood volume and is not related to exercise intensity [22]. Given the advantages (non-invasive, lightweight, portable, waterproof and suitable for outdoor training), NIRS technology provides important information about muscle response to exercise as well as muscle recovery and, in view of this, was used in our research.

In addition, the authors did not encounter an article in the literature on the effect of ashwagandha administration on the local capacity of muscles to utilize oxygen. For this reason, a study was undertaken to characterize changes in aerobic capacity, muscle oxygenation and haematological parameters in the blood under the influence of 8-week HIIT in healthy male non-athletes.

In most studies evaluating the effects of ashwagandha supplementation on health parameters, the dose used was 300 mg of the extract per day, which is the dose recommended for people with sedentary lifestyles. For physically active people/athletes, a dose of 600 mg per day is recommended and this was the dose used in most studies evaluating the effects of ashwagandha on physical performance [1]. Although there are studies in the scientific literature reporting higher doses (750 or 1000 mg per day) [1], in the current study we chose a dose of 600 mg per day, taking into account the safety of administration supported by the results of most studies [1, 3], the recommendations of the supplement manufacturer and in accordance with the national guidelines (Dietary Supplement Panel Resolution) for the maximum recommended daily intake of the product.

## MATERIALS AND METHODS

### Participants

The study included healthy male participants. Exclusion criteria were: practicing high-performance sports, the use of tobacco products, alcohol consumption, taking any medications that increase physical performance, as well as drugs/medications that are sedative, antianxiety or sleep-inducing; orthopedic injury or past surgery within the last 6 months; chronic diseases; known intolerance of herbal supplements of similar composition; taking any herbal preparations or supplements containing antioxidant and anti-inflammatory substances within the last 3 months. The participants were recruited from the students of Faculty of Physical Education and Health in Biała Podlaska.

Before the experiment, participants were randomly assigned, in a double-blind fashion, to two groups. Initially, 41 students met the inclusion criteria and were randomly assigned to either the ashwagandha (n = 20) or placebo (n = 21) group. The R programme was used for the randomisation procedure [23]. Three participants (two from the ashwagandha group and one from the placebo group) dropped out of the study for personal reasons. Due to technical problems with measuring muscle oxygenation during the maximal graded exercise test, five students were excluded from the analysis due to incomplete data. Finally, 33 students (17 from the ashwagandha group and 16 from the placebo group) were included in the statistical analysis in this manuscript.

The study was conducted following the principles of the Declaration of Helsinki. All participants gave their consent to participate in the study and the research protocol was approved by the Local Ethics Committee of the Józef Piłsudski University of Physical Education in Warsaw (SKE 01-43/2022). Students were asked to refrain from modification of their diet during the study period.

### Supplementation

Two capsules of ashwagandha extract were administered daily (2 × 300 mg per day) for a period of 8 weeks. One capsule contained 300 mg of ashwagandha root extract, KSM-66, standardized to a 5 % concentration of withanolides as measured by HPLC.

Placebo capsules were administered in the same form as ashwagandha supplement. Both the ashwagandha and placebo capsules (gelatin) were identical in appearance (size, shape, and color), and they both were manufactured by the same producer. The placebo capsules contained microcrystalline cellulose as an inert filler, magnesium salts of fatty acids, silicon dioxide (stored in bottles, which previously contained ashwaganda extract, so that the odor of ashwagandha permeated to the placebo capsules). The subjects were instructed to take the capsules twice a day after meals (breakfast and dinner). The capsules were supplied to the students weekly in dark nontransparent bottles. The participants (from both placebo and ashwagandha group) declared good tolerability of capsules, with no adverse events.

### Blood sampling and hematological analyses

Blood samples from the ulnar vein were obtained in the morning (at 7:00 a.m.), after an overnight fast, prior to (pre) and after (post) an 8-week HIIT and supplementation (in resting condition; at least 48 hours after the last training session). The blood samples were collected to a tube (2 ml) with anticoagulant (EDTA) to hematological measurements. Hematological parameters were assessed immediately after blood collection, using an automated method at a local commercial diagnostic laboratory (hematology analyzer; BIOMAXIMA BM HEM 5 TS; Poland). These parameters included hemoglobin (Hb), hematocrit (Ht), red blood cells (RBC), leukocyte (LEU) and subset counts: lymphocytes (LYMPH), monocytes (MONO), total granulocytes (GR), eosinophils (EOS), basophils (BASO), as well as platelets (PLT).

### Exercise performance and HIIT program

Before and after the training period (at least 48 hours after the last training session), the students completed an incremental rowing ergometer test (Concept 2 rowing ergometer) to volitional fatigue (i.e., with a gradual increase in intensity until the subjects had to stop due to exhaustion, GXT Test). The damper was set to 4–6. The test consisted of several 3-minute trials with 1-minute passive rest periods between trials (for collection of capillary blood samples). The power used in the first trial was 100 W, which increased by 30 W in each subsequent trial.

During the test, heart rate (HR) was continuously recorded using the H9 Heart Rate Sensor (Polar Electro Oy, Finland). Gas exchange was measured during each testing session using wearable and wireless breath-by-breath pulmonary gas analyzer (MetaLyzer 3B, Cortex Biophysik GmbH, Germany). Lactate concentration (LA) was measured in capillary blood, collected from the fingertip before the GXT, immediately after the subjects completed each 3-minute trial and 3 minutes after the entire test was performed, using the Super GL2 device (Dr Müller, Germany).

The following physiological parameters, as recognized indicators of aerobic exercise performance, was used to assess the impact of training on the physical condition of the subjects: 1) maximal oxygen uptake (${\dot{\text{V}}\text{O}}_{2\max}$, in absolute and relative values); 2) time to exhaustion (test time); 3) maximal aerobic power (MAP, average power output of final load; and 4) anaerobic threshold (P_AT4_ absolute and relative), i.e. the power at blood lactate concentration 4 mmol/l.

Eight-week HIIT was performed on a Concept 2 rowing ergometer (Morrisville, NC) under the supervision of qualified instructors. The initial incremental rowing ergometer test to volitional fatigue (performed prior to the exercise program) was preceded by 2–3 introductory sessions aimed at developing proper rowing technique.

The indexed training consisted of 3 sessions per week, with at least 1 day of rest between sessions. During the first two weeks (weeks 1–2), the training was designed to prepare participants for high-intensity efforts, so the load was 75–80% of the individual’s HRmax, measured during an incremental rowing test (5 sets of 1.5 minutes). The HIIT training programme in the following weeks followed the pattern: 5 sets of 1.5 minutes at 85% MAP (weeks 3–4), 6 sets of 1.5 minutes at 90% MAP (weeks 5–6), 7 sets of 1.5 minutes at 95% MAP (weeks 7–8) (Table 1). In all workouts (weeks 1–8), the rest intervals between sets were equal to the duration of the exercise (1:1) with 70 W power output. Each workout was preceded by a warm-up and ended with a cooling down. The warm-up consisted of 5-min continuous rowing at an intensity equivalent to a HR of 130–140 beats/min, while the cooling down involved rowing for about 3 minutes at low power (about 50 W). The HIIT training program was developed based on the work of Driller et al. [24] with modifications.

**TABLE 1:** The HIIT training programme in the following weeks.

Weeks 1–2	Weeks 3–4	Weeks 5–6	Weeks 7–8
5 sets of 1.5 min. at 75–80% of HRmax	5 sets of 1.5 min. at 85% MAP	6 sets of 1.5 min. at 90% MAP	7 sets of 1.5 min. at 95% MAP

Abbreviations: HRmax – heart rate maximum; MAP – maximal aerobic power.

### Anaerobic threshold and Maximal Aerobic Power

Anaerobic threshold (4 mmol, P_AT4_) were determined based on the application described by Newell et al. [25]. Maximal aerobic power (MAP) was calculated as a proportion of the time and a power of the last executed bout in the GXT [26]. The equation for calculation is presented below:


$$\text{MAP }={\text{M}}_{\text{LFE}}+({\text{T}}_{\text{LE}}/{\text{T}}_{\text{ST}}*\Delta \text{P}$$

MAP – maximal aerobic power; M_LFE_ – power of the last fully executed step; T_LE_ – time executed in the final step; T_St_ – time of the last step; ΔP – increase in power between last two steps.

### Maximal Oxygen Consumption

Maximal oxygen uptake (${\dot{\text{V}}\text{O}}_{2\max}$) was defined as the highest amount of oxygen consumption over a 30-s period during the test. The maximal intensity of exercise necessary for the estimation of ${\dot{\text{V}}\text{O}}_{2\max}$ was defined by the following criteria: the VO_2_ plateauing with increasing workload, the post-exercise blood lactate concentration > 8 mmol/l, the respiratory exchange ratio (RER) > 1.1, and attainment of the age-adjusted maximal heart rate expressed as HRmax = 220–participant age. If at least two of the above criteria were met during the exercise, the attained effort and oxygen uptake were considered maximal.

### Measurements of muscle oxygen saturation

**FIG. 1:**
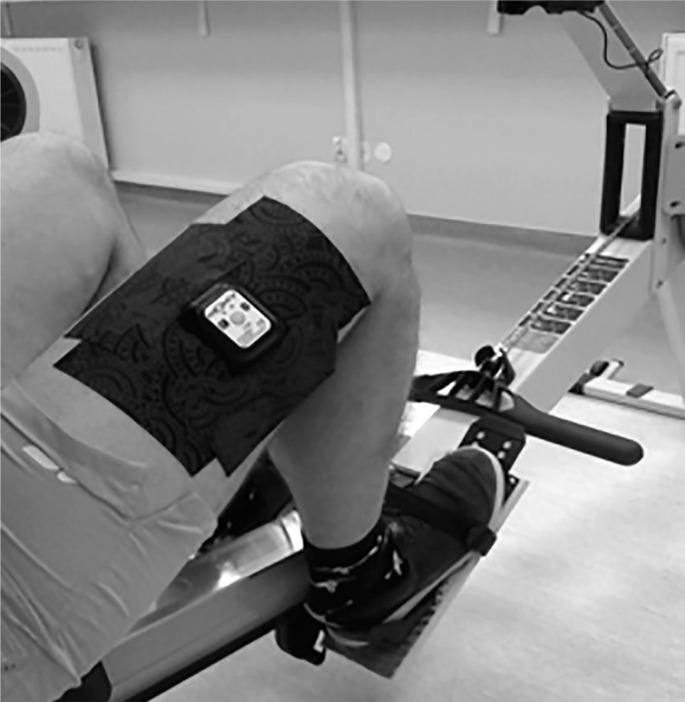
The location of the MOXY monitor on the vastus lateralis muscle belly.

During GXT test, a NIRS device (Moxy Monitor; Fortiori Design LLC, Hutchinson, MN, USA) was placed on the vastus lateralis (VL) muscle that is active during rowing. Figure 1 shows the placement of the NIRS monitor.

The Moxy monitor is a continuous wave near-infrared spectroscopy monitor. It uses a new type of algorithm that is based on Monte Carlo modeling. The system uses 4 wavelengths at 680, 720, 760, and 800 nm. It has 2 emitters to detector spacings of 12.5 and 25 mm. The device was placed approximately 15 ± 2 cm above the proximal border of the patella on the vastus lateralis muscle belly and was fixed to the right limb with a dark 7.5 cm dynamic tape by the same person. In students, skinfold was measured at the site where the Moxy Monitor was placed. It should be emphasized that the NIRS signal is affected by the thickness of subcutaneous adipose tissue thickness (ATT), e.g., 5 mm thickness reduces the penetration of infrared light by about 20% [27, 28].

**FIG. 2:**
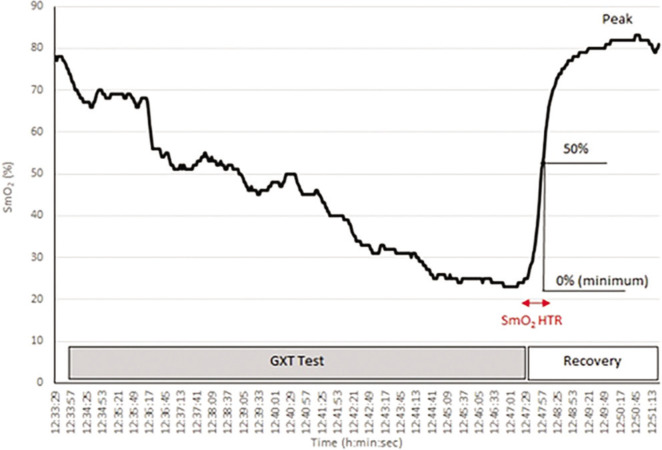
Method of evaluating the time for 50% recovery muscle oxygen saturation (SmO2 Recovery Half Time, SmO2HTR) after the maximal graded exercise test (GXT) for one of the male students (compiled according to Nagasawa 2013 [30])

The SmO_2_ was recorded during exercise and recovery. SmO_2_ reflects the dynamic balance between oxygen (O_2_) consumption and supply [29]. The differences in SmO_2_ (Δ SmO_2_) between maximal and minimal exercise levels were also calculated. The reoxygenation rate after GXT test were evaluated as the 50% time required for SmO_2_ recovery (SmO_2_ Recovery Half Time, SmO_2_HTR) [30, 31], Fig. 2.

The SmO_2_ data values for further calculations were averaged from measurements made during 2 s at exercise minimum (SmO_2_ Min) and recovery maximum (SmO_2_ Max). The mean SmO_2_ for 2 seconds immediately after the completion of the exercise was defined as 0% and the maximum SmO_2_ in the first 3 min of the recovery phase after completion of exercise was defined as 100%. The SmO_2_ Δ Time was then defined as the time from the completion of exercise to the time to reach 50% SmO_2_Max. SmO_2_ Reoxy Rate was calculated according to the following formula (18):


$${\text{SmO}}_{2}\text{ Reoxy Rate }(\%/\text{s})=50\%\;{\text{SmO}}_{\text{2}}\text{Max }(\%)/{\text{SmO}}_{\text{2}}\;\Delta \text{ Time}(\text{s})$$

SmO_2_ threshold values were determined by averaging 30-s intervals for the respective exercise loads.

### Statistical analysis

Statistical analysis was conducted with Statistica version 13.3 (Stat-Soft, Krakow, Poland). Normality assumptions were checked on all variables using Shapiro–Wilk test and visual inspection (quantile distribution plots). Also, homogeneity of variance was checked with Levene’s test. All values were reported as mean ± standard deviation (SD). The level of statistical significance was set at p < 0.05. Statistical significance of intergroup (ashwagandha vs. placebo) differences in anthropometric characteristics (age, height, etc.) were verified with unpaired Student t-test. The two-way ANOVA for repeated measurements: 2 groups (placebo, ashwagandha) × 2 time points (pre-, post-) was applied for comparison of muscle oxygenation and the performance results obtained in the maximal graded exercise test. For detailed comparisons (between and within groups) the post-hoc Tukey´s test for unequal samples was utilised. Furthermore, the effect size for the ANOVA was estimated (eta squared; η^2^). The same statistical methods were used for haematological blood indices, however, after logarithmic transformation (natural logarithm). Sample size was calculated using GPower version 3.1 [32]. With the assumptions: effect size f = 0.25, α = 0.05, power 0.8, required sample size amounted 34 for two-way ANOVA with repeated measurements (two time points). The power for the group of 33 participants in our study taken for analysis was 0.79.

## RESULTS

The characteristics of the subjects are shown in Table 2. In terms of the anthropometric indices analyzed, as well as maximum oxygen uptake at the beginning of the study, there were no statistically significant differences between the study groups (P > 0.05).

**TABLE 2:** Basic characteristics of the examined group of students (mean ± SD).

Variable	Ashwagandha (n = 17)	Placebo (n = 16)
Age (years)	20.3 ± 0.8	20.8 ± 1.8
Body height (cm)	181.9 ± 6.3	182.8 ± 7.6
Body weight (kg)	81.2 ± 9.6	79.2 ± 9.5
BMI (kg/m^2^)	24.6 ± 2.7	23.7 ± 1.8
${\dot{\text{V}}\text{O}}_{2\max}$(ml/kg/min)	46.1 ± 6.7	46.9 ± 4.5

No inter-group differences were found at p < 0.05 (unpaired Student t-test).

**FIG. 3:**
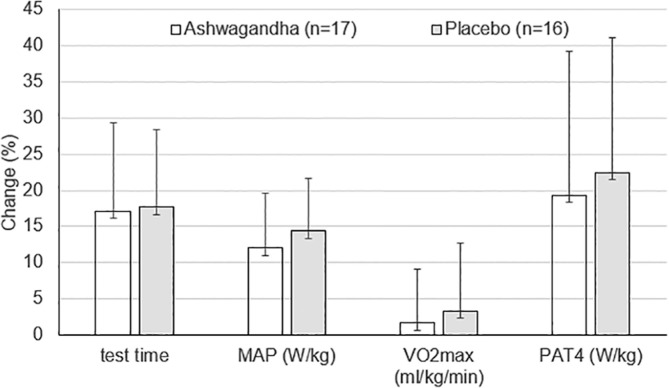
Percentage changes in test time, maximal aerobic power (MAP) (W/kg), maximal oxygen uptake (VO2max) (ml/kg/min) and power at anaerobic threshold (PAT4) (W/kg) after 8-week HIIT training combined with ashwagandha supplementation or placebo. Values are mean ± SD No inter-group differences were found at p < 0.05 (unpaired Student t-test).

The mean values of selected indices of exercise capacity are shown in Table 3. No effects of group or time × group interaction were shown in any of the variables analyzed. In turn, main effect of time was found for test time (P = 0.00001; η^2^ = 0.77), MAP and P_AT4_ (in both absolute and relative values; P = 0.00001; with η^2^ amounted 0.60–0.78), as well as in ${\dot{\text{V}}\text{O}}_{2\max}$ (in absolute values; P = 0.019; η^2^ = 0.16). In the parameters listed above, except for ${\dot{\text{V}}\text{O}}_{2\max}$, post-hoc analysis showed within-group differences (pre-post) for both placebo (P < 0.001) and ashwagandha groups (P < 0.001). There were no significant differences between groups in the percentage changes in exercise capacity parameters (Fig. 3).

**TABLE 3:** Values of selected indices recorded during the maximal graded exercise test performed before (pre) and after (post) 8-week HIIT training combined with ashwagandha (n = 17) or placebo (n = 16) supplementation.

Variable/Group	Time	Ashwagandha (n = 17)	Placebo (n = 16)	Main effects: P-Values (η^2^, Effect Size)		

				Time	Group	Time × Group
Test time (min:s)	Pre	15:14 ± 3:31	15:39 ± 3:33	0.00001	0.60	0.47
	Post	17:27 ± 2:42^***^	18:13 ± 3:31^***^	(0.77)	(0.009)	(0.02)

MAP (W)	Pre	226 ± 36	230 ± 51	0.00001	0.64	0.38
	Post	252 ± 32^***^	261 ± 49^***^	(0.78)	(0.007)	(0.02)

MAP (W/kg)	Pre	2.81 ± 0.49	2.90 ± 0.46	0.00001	0.41	0.28
	Post	3.13 ± 0.46^***^	3.29 ± 0.41^***^	(0.77)	(0.02)	(0.04)

${\dot{\text{V}}\text{O}}_{2\max}$ (l/min)	Pre	3.65 ± 0.58	3.66 ± 0.61	0.019	0.84	0.66
	Post	3.77 ± 0.41	3.84 ± 0.68	(0.16)	(0.001)	(0.006)

${\dot{\text{V}}\text{O}}_{2\max}$ (ml/kg/min)	Pre	46.1 ± 6.7	46.9 ± 4.5	0.15	0.54	0.55
	Post	46.7 ± 6.2	48.3 ± 5.5	(0.06)	(0.01)	(0.01)

LA_PEAK_ (mmol/l)	Pre	12.3 ± 2.6	12.1 ± 2.8	0.43	0.82	0.31
	Post	12.2 ± 1.6	12.8 ± 2.7	(0.02)	(0.002)	(0.03)

P_AT4_ (W)	Pre	151 ± 38	150 ± 44	0.00001	0.87	0.52
	Post	176 ± 35^***^	181 ± 41^***^	(0.60)	(0.0008)	(0.01)

P_AT4_ (W/kg)	Pre	1.86 ± 0.47	1.89 ± 0.42	0.00001	0.67	0.44
	Post	2.17 ± 0.42^***^	2.27 ± 0.39^***^	(0.61)	(0.006)	(0.02)

Values are mean ± SD.

*** – difference (p < 0.001) between pre and post values within the same group (asphagandha or placebo). Abbreviations: MAP – maximal aerobic power;

LA_PEAK_ – post-exercise lactate concentration in the blood; P_AT4_ – power at anaerobic threshold (lactate = 4 mmol/l); η^2^ – *eta squared* (*Effect Size)*.

Data on muscle oxygenation parameters recorded during the maximal graded exercise test are shown in Table 4. Statistical analysis revealed a significant time and group interaction for SmO_2_Min (P = 0.026; η^2^ = 0.15) and ΔSmO_2_ (P = 0.011; η^2^ = 0.19), with post-hoc analyses revealed significant pre-post differences in the placebo group (i.e. a decrease in SmO_2_Min, P < 0.05; and an increase in ΔSmO_2_, P < 0.01), whereas no changes were found in the ashwagandha group (P > 0.05). In turn, post-hoc analysis showed no significant intergroup differences in the above-mentioned parameters (SmO_2_Min and ΔSmO_2_), for both pre (P > 0.05) and post intervention (P > 0.05). Moreover, a main time effect was found for SmO_2_Max (P = 0.016; η^2^ = 0.17), and ΔSmO_2_ (P = 0.0039; η^2^ = 0.24).

**TABLE 4:** Values of selected indices of muscle oxygenation recorded during the maximal graded exercise test performed before (pre) and after (post) 8-week HIIT training combined with ashwagandha (n = 17) or placebo (n = 16) supplementation.

Variable/Group	Time	Ashwagandha (n = 17)	Placebo (n = 16)	Main effects: P-Values (η^2^,Effect Size)		
							Time	Group	Time × Group
SmO_2_Max (%)	Pre	81.9 ± 6.1	79.1 ± 8.2	0.016	0.33	0.33
	Post	83.6 ± 4.4	82.9 ± 4.3	(0.17)	(0.03)	(0.03)

SmO_2_Min (%)	Pre	18.1 ± 13.1	17.8 ± 6.7	0.097	0.24	0.026
	Post	19.1 ± 13.7	11.4 ± 6.1^*^	(0.09)	(0.04)	(0.15)

ΔSmO_2_(%)	Pre	63.7 ± 15.4	61.3 ± 10.7	0.0039	0.52	0.011
	Post	64.5 ± 12.7	71.7 ± 6.0^**^	(0.24)	(0.01)	(0.19)

SmO_2_HTR(s)	Pre	26.6 ± 4.8	25.1 ± 10.1	0.57	0.44	0.85
	Post	25.4 ± 5.1	24.5 ± 4.0	(0.01)	(0.02)	(0.001)

SmO_2_ ReoxyRate (%/s)	Pre	0.79 ± 0.16	0.88 ± 0.27	0.56	0.32	0.47
	Post	0.85 ± 0.17	0.87 ± 0.17	(0.01)	(0.03)	(0.02)

Values are mean ± SD. ^*, **^– significant difference (p < 0.05 and p < 0.01, respectively) between pre and post values within the same group (ashwagandha or placebo). Abbreviations: SmO_2_ Max – maximum muscle oxygenation; SmO_2_ Min – minimum muscle oxygenation; ΔS mO_2_ (%) – SmO_2_ Max minus SmO_2_ Min; SmO_2_HTR(s) – the time from the completion of exercise to reach 50% SmO_2_Max; SmO_2_ ReoxyRate(%/s) – recovery rate of muscle oxygenation; η^2^ – *eta squared* (*Effect Size*).

Mean values of haematological parameters are shown in table 5. No significant main effects (i.e. time, group) and time and group interaction were found for most of these parameters (except for EOS (10 × 3/µl) and MONO (%)). A main effect of time (P = 0.03) was found for EOS (in absolute values), with post-hoc analysis showing only an increasing trend for the placebo group (P = 0.1), but not for the ashwagandha group (P = 0.8). Regarding MONO in relative terms (%), despite a significant main effect of time (P = 0.04), no significant post-hoc analyses were found in both ashwagandha (P = 0.7) and placebo (P = 0.3) groups. Furthermore, in both groups and at both measurement periods (before and after the 8-week study period), the mean values of all haematological parameters were within the reference range for the study population.

**TABLE 5:** Haematological blood indices of students before (pre) and after (post) 8-week high intense interval training (HIIT) combined with ashwagandha supplementation (n = 17) or placebo (n = 16).

Variable/Group	Reference range	Time	Ashwagandha (n = 17)	Placebo (n = 16)	Main effects		

					Time	Group	Time × Group
WBC (10 × 3/µl]	4.2–9.07	Pre	7.17 ± 2.24	6.07 ± 1.02	0.59	0.15	0.20
		Post	6.97 ± 1.23	6.56 ± 1.75			

LYMPH (10 × 3/µl)	1.0–3.7	Pre	2.68 ± 0.86	2.33 ± 0.70	0.32	0.21	0.81
		Post	2.76 ± 0.89	2.46 ± 0.60			

MONO (10 × 3/µl)	0.0–0.9	Pre	0.55 ± 0.22	0.53 ± 0.16	0.14	0.97	0.53
		Post	0.47 ± 0.1	50.49 ± 0.24			

GR (10 × 3/µl)	1.5–7.0	Pre	3.66 ± 1.96	2.97 ± 0.57	0.84	0.29	0.27
		Post	3.44 ± 0.90	3.29 ± 1.41			

EOS (10 × 3/µl)	0.0–0.4	Pre	0.25 ± 0.15	0.22 ± 0.14	0.03	0.84	0.28
		Post	0.27 ± 0.15	0.28 ± 0.21			

BASO (10 × 3/µl)	0.0–0.2	Pre	0.04 ± 0.01	0.03 ± 0.02	0.19	0.35	0.39
		Post	0.04 ± 0.01	0.04 ± 0.01			

LYMPH (%)	20.0–45.0	Pre	38.81 ± 10.11	37.80 ± 6.66	0.60	0.74	0.93
		Post	39.44 ± 8.83	38.66 ± 8.97			

MONO (%)	3.0–12.0	Pre	7.73 ± 1.93	9.03 ± 3.64	0.04	0.22	0.60
		Post	6.87 ± 1.99	7.56 ± 3.08			

GR (%)	45.0–70.0	Pre	49.36 ± 11.32	48.97 ± 6.24	0. 49	0.95	0.88
		Post	49.26 ± 8.92	49.29 ± 8.77			

EOS (%)	1.0–5.0	Pre	3.46 ± 1.76	3.63 ± 2.33	0.12	0.79	0.94
		Post	3.84 ± 1.86	4.04 ± 2.39			

BASO (%)	0.0–2.0	Pre	0.59 ± 0.16	0.57 ± 0.27	0.47	0.96	0.47
		Post	0.59 ± 0.19	0.63 ± 0.22			

RBC (10 × 3/µl)	4.5–5.9	Pre	5.16 ± 0.38	5.14 ± 0.26	0.11	0.60	0.31
		Post	5.14 ± 0.35	5.05 ± 0.23			

Hb (g/dl)	13.5–17.5	Pre	15.58 ± 1.28	15.19 ± 0.84	0.12	0.24	0.64
		Post	15.45 ± 1.35	14.96 ± 0.74			

HCT (%)	39.0–53.0	Pre	45.69 ± 3.44	45.01 ± 2.12	0.11	0.31	0.33
		Post	45.48 ± 3.70	44.15 ± 1.94			

PLT (10 × 3/µl)	150–350	Pre	227.7 ± 37.6	220.8 ± 50.9	0.76	0.76	0.63
		Post	226.9 ± 44.1	224.5 ± 50.2			

Values are mean ± SD. No significant differences were seen between term 1 and term 2 (within the same group) or between groups (within the same term: 1 or 2) (P > 0.05). Abbreviations: WBC: white blood cell count; LYMPH: lymphocytes; MONO: monocytes; GR: granulocytes; EO: eosinophils; BASO: basophils; RBC: red blood cell count; Hb: haemoglobin; Hct: hematocrit; PLT: platelet count.

## DISCUSSION

The main finding of our study is that ashwagandha supplementation does not seem to affect training-induced improvements in aerobic capacity in healthy men. Despite significant increases in aerobic performance indices (test time, MAP and P_AT4_ in absolute and relative values) under 8-week HIIT, there was no greater improvements in the ashwagandha group compared to the placebo group (Table 3). The observed changes in the aforementioned indices of aerobic capacity, such as post-training increases in test time (by 17.1 ± 12.3 and 17.7 ± 10.7%), as well as MAP in relative units (by 12.1 ± 7.6 and 14.4 ± 7.2%) and P_AT4_ in relative units (by 19.3 ± 19.9 and 22.5 ± 18.6%) in the ashwagandha and placebo groups, respectively, indicate that HIIT is highly effective in improving aerobic capacity using the rowing ergometer (Fig. 3).

Our finding did not confirm the previous results of a meta-analysis based on five studies conducted in the Indian population indicating improvements in aerobic capacity after 4–12 weeks of ashwagandha supplementation (at a dose of 300–1000 mg per day), both in untrained and trained individuals [1, 3]. However, it should be mentioned that two of these five papers [2, 33] used indirect methods, including the Cooper test, to calculate ${\dot{\text{V}}\text{O}}_{2\max}$. In turn, two other papers [34, 35], lacked any data on the method of measuring ${\dot{\text{V}}\text{O}}_{2\max}$. Also, for a recent paper on the effects of ashwagandha on aerobic capacity [36], which was not included in the meta-analysis by Bonilla et al. [1], ${\dot{\text{V}}\text{O}}_{2\max}$ was calculated indirectly, using the Cooper test. In only one of the papers included in the meta-analysis performed by Bonilla et al. [1], an aerobic capacity index was determined directly in a graded exercise test performed on a treadmill [37]. In the above study, the authors found significant improvements in ${\dot{\text{V}}\text{O}}_{2\max}$ and time to exhaustion in elite Indian cyclists as a result of 8-week supplementation with 500 mg of ashwagandha root aqueous capsules twice daily [37]. However, it should be noted that the cyclists’ mean baseline ${\dot{\text{V}}\text{O}}_{2\max}$ level (45.5 ml / kg /min) was comparable to that of our non-athlete students (Table 3). In addition, there was no information regarding the monitoring of cyclists’ training. A strength of our work is that we used a reliable method to assess aerobic capacity and the 8-week training was fully supervised, with the training loads (in relative terms, i.e., per kilogram of body weight) being identical for each subject. In addition, prior to the proper GXT (performed before the exercise programme), we implemented 2–3 introductory sessions aimed at learning correct rowing technique. Furthermore, our study involved a homogeneous population of healthy men. On the other hand, it could not be excluded that the response to ashwagandha supplementation may be genetically/environmentally determined (with possible inter-ethnic/racial differences). It is also possible that a higher supplement dose (i.e. 1000 mg per day, as in Shenoy’s study, instead of 600 mg per day, as in our study) is needed to achieve improvement in exercise performance. Also, it cannot be excluded that a longer period of ashwagandha supplementation may be needed to exert benefits in healthy population engaged in intense training regimen. Further research is warranted to address all of the issues mentioned above.

In order to test the potential mechanisms responsible for the improvement in aerobic capacity as a result of ashwagandha supplementation, as described in previous papers [1, 3], muscle oxygenation parameters were monitored during GXT in our study. In the available literature there are relatively few reports evaluating the effect of training on the characteristics of changes in muscle oxygenation indices [18, 20, 38], and the observation above is particularly applicable to HIIT [19]. The last cited study documented the effects of four various types of HIIT on changes in muscle oxygenation indices in sprint canoe-kayak athletes. Interestingly in our own research, 8-week HIIT alone appears to affect changes in SmO_2_ Max and ΔSmO_2_ (significant main effect of time), as well as SmO_2_Min (a tendency toward a time effect). However, contrary to expectations, significant changes in ΔSmO_2_ and SmO_2_Min after the 8-week study period were observed in the placebo group, but not in the ashwagandha group, with significant interaction of time and group for SmO_2_Min and ΔSmO_2_ (Table 4). Namely, only in the placebo group was there an improvement in the degree of oxygen utilization by working muscles (ΔSmO_2_) after 8-week training, which was mainly due to a decrease in minimum exercise SmO_2_ (Table 4). Generally, the lowest muscle O_2_ levels (SmO_2_Min) is considered to be indirectly related to exercise intensity (muscle O_2_ consumption) (39, 40). Thus, lower SmO_2_Min in the placebo group after training than before training may have been related to higher intensity of the test performed post-training (higher O_2_ consumption), as compared to pre-training. On the other hand, our blood lactate results do not seem to support this hypothesis, as there were no significant differences in blood LApeak (as a marker of exercise intensity). However, it should be noted that in the placebo group there was a slight trend towards higher LApeak after HIIT training (12.8 ± 2.7 mmol/l) compared to baseline (12.1 ± 2.8 mmol/l), Table 3. Such a response was not observed in the ashwagandha group (12.3 ± 2.6 vs 12.2 ± 1.6 mmol/l, respectively). On the other hand, the lack of any significant differences in LApeak cannot exclude the possibility that, after 8-week training (compared to the pre-training condition), individuals in the placebo group may have performed the test at a higher intensity (with more intensified anaerobic glycolysis and muscle lactate production). Undoubtedly, additional information could be provided by the determination of blood acid-base balance indices, which can be considered as some limitations of this study. On the other hand, it is difficult to explain why these muscle oxygenation changes apply to the placebo but not to ashwagandha group, even suggesting some blunting effect of ashwagandha on training-induced improvements in muscle oxygenation. These issues need to be explained in further study.

Finally, although increased muscle oxygen utilization was observed only in the placebo group, improvements in maximal oxygen uptake in absolute terms occurred in both groups (by 4.9 ± 15.3% and 4.8 ± 6.8% in the ashwagandha and placebo group, respectively), Table 3. However, in relative units, ${\dot{\text{V}}\text{O}}_{2\max}$ did not improve significantly over the period (by 1.7 ± 7.4% in the ashwagandha group and 3.3 ± 9.4% in the placebo group) as a result of an increase in post-training body weight by an average of 1.5 kg in both groups, Table 3, Fig.3. The data obtained confirmed the lack of a beneficial effect of ashwagandha supplementation on exercise oxygen uptake and utilization. In the study Paquette et al. [19], short interval training sessions HIIT-15 s and HIIT – 30 s (both with 110% peak power output from Maximal Incremental Test performed on a canoe-kayak ergometer) elicited the longest time near ${\dot{\text{V}}\text{O}}_{2\max}$, potentially conductive to ${\dot{\text{V}}\text{O}}_{2\max}$ improvements. The conclusions presented from the cited studies, may contribute to explaining the lack of significant changes in relative ${\dot{\text{V}}\text{O}}_{2\max}$ in our study, when the load duration was longer (HIIT – 90 s) at a lower intensity (from 75 to 80% HRmax in weeks 1–2 and 85 to 95% MAP in 3–8 weeks).

In addition, the recovery rate indices (SmO_2_HTR and SmO_2_ ReoxyRate) also showed no significant changes in the two groups after training, Table 4. Importantly, in our previous study, the rate of SmO_2_ reoxygenation showed a significant correlation with ${\dot{\text{V}}\text{O}}_{2\max}$ and exercise capacity in GXT [41]. In speed skaters with higher maximal oxygen uptake and test duration, the quadriceps muscle oxygenation returned to maximal levels faster after GXT. Similarly Ichimura et al. [29] also showed that the rate of muscle reoxygenation after a ramp test was faster in subjects with higher ${\dot{\text{V}}\text{O}}_{2\max}$ levels. These results suggest that the rate of muscle reoxygenation in the graded exercise test depended on the aerobic capacity of speed skaters.

In contrast to the cited studies, in both groups of the current study, the 8-week training period did not increase the rate of SmO_2_ recovery. It can be speculated that this was too short a training period to affect, in addition to improvements in test duration, MAP and PAT4, other adaptive changes typically associated with endurance training. These included an improvement in muscle fiber capillarization, an increase in the total number of mitochondria and mitochondrial size, as well as an increase in oxidative enzyme activity and myoglobin content [42]. Indeed, the aforementioned morphological and metabolic changes in skeletal muscle are associated with improved recovery rates, and the relatively short training period in our study was likely insufficient to induce them.

The conclusion that there is no beneficial effect of ashwagandha administration on aerobic exercise capacity is further strengthened by the observed lack of differences between the study groups in terms of anaerobic threshold power, Table 3. In contrast to maximal oxygen uptake, exercise indices at the anaerobic threshold level are generally considered more sensitive to training (compare Fig. 3). However, again, no more favorable threshold power results were obtained in the ashwagandha group, as compared to the placebo group.

On the other hand, as already mentioned, potential effect of ashwagandha supplementation on the parameters of muscle oxygenation and their changes induced by training need to further explanation, as the results obtained are difficult to compare due to the lack of studies with ashwagandha supplementation using the discussed methods of analyzing muscle oxygenation under exercise conditions.

The possible beneficial effects of ashwagandha on aerobic capacity may be mediated through red blood cell indices (RBC, Hb, Ht) as reported in a recent review study [43]. However, these results refer to studies conducted without a placebo group [44] or from studies whose results need to be confirmed due to the lack of significant intergroup differences [2]. Our double-blind placebo-controlled study did not confirm the above-mentioned observations, given the non-significant time and group interaction in haematological indices (Table 5). As mentioned above, it cannot be excluded that higher dose of ashwagandha might be needed to exert beneficial effects in healthy young men. In the study of Raut et al. [44], increasing daily dosage of ashwagandha extract every 10 days was used (750 mg/day × 10 days, 1000 mg/day × 10 days, 1250 mg/day × 10 days) in healthy volunteers, but without any training program. On the other hand, the supplementation regimen used in the study by Malik et al [2] in young hockey players was similar to that in our study (500 mg daily for 8 weeks). Our findings regarding the lack of effect of ashwagandha on haematological indices are consistent with the results reported by Lopresti et al. [45], although his study involved the intake of ashwagandha extract (at the same dose as in our study, i.e. 2 × 300 mg daily for 8 weeks) in mildly anxious, healthy overweight men aged 40–70 years. Also in our previous study on professional wrestlers [46], we found no effect of ashwagandha supplementation (2 × 300 mg/day for 8 weeks) on haematological indices. Moreover, no significant changes in haematological indices were found in the study of Ziegenfuss et al. [47] after 12-week ashwagandha supplementation (500 mg dose daily) in recreationally active young underwent strength training. Precisely, beneficial effects was seen rather in placebo instead in ashwagandha group, i.e. the increase in RBC, Hb and Ht [47]. Therefore, further research is needed to resolve these discrepancies in results regarding the effects of ashwagandha on aerobic capacity and the mechanisms responsible for these effects.

## CONCLUSIONS

In conclusion, in healthy men subjected to an 8-week HIIT, ashwagandha supplementation does not appear to affect haematological status or offer additional benefits in aerobic capacity over those observed under training. Moreover, the inconclusive effect of ashwagandha on training-induced changes in muscle oxygenation needs to be verified in further studies.
